# Neuroprotective Effect of TAT-14-3-3ε Fusion Protein against Cerebral Ischemia/Reperfusion Injury in Rats

**DOI:** 10.1371/journal.pone.0093334

**Published:** 2014-03-26

**Authors:** Yuanjun Zhu, Qixin Bu, Xiaoyan Liu, Wenhui Hu, Yinye Wang

**Affiliations:** 1 Department of Molecular and Cellular Pharmacology, School of Pharmaceutical Sciences, Peking University, Beijing, China; 2 Department of Neuroscience, Temple University School of Medicine, Philadelphia, Pennsylvania, United States of America; Universidad de Castilla-La Mancha, Spain

## Abstract

Stroke is the major cause of death and disability worldwide, and the thrombolytic therapy currently available was unsatisfactory. 14-3-3ε is a well characterized member of 14-3-3 family, and has been reported to protect neurons against apoptosis in cerebral ischemia. However, it cannot transverse blood brain barrier (BBB) due to its large size. A protein transduction domain (PTD) of HIV TAT protein, is capable of delivering a large variety of proteins into the brain. In this study, we generated a fusion protein TAT-14-3-3ε, and evaluated its potential neuroprotective effect in rat focal ischemia/reperfusion (I/R) model. Western blot analysis validated the efficient transduction of TAT-14-3-3ε fusion protein into brain via a route of intravenous injection. TAT-14-3-3ε pre-treatment 2 h before ischemia significantly reduced cerebral infarction volume and improved neurologic score, while post-treatment 2 h after ischemia was less effective. Importantly, pre- or post-ischemic treatment with TAT-14-3-3ε significantly increased the number of surviving neurons as determined by Nissl staining, and attenuated I/R-induced neuronal apoptosis as showed by the decrease in apoptotic cell numbers and the inhibition of caspase-3 activity. Moreover, the introduction of 14-3-3ε into brain by TAT-mediated delivering reduced the formation of autophagosome, attenuated LC3B-II upregulation and reversed p62 downregulation induced by ischemic injury. Such inhibition of autophagy was reversed by treatment with an autophagy inducer rapamycin (RAP), which also attenuated the neuroprotective effect of TAT-14-3-3ε. Conversely, autophagy inhibitor 3-methyladenine (3-MA) inhibited I/R-induced the increase in autophagic activity, and attenuated I/R-induced brain infarct. These results suggest that TAT-14-3-3ε can be efficiently transduced into brain and exert significantly protective effect against brain ischemic injury through inhibiting neuronal apoptosis and autophagic activation.

## Introduction

Stroke is responsible for more than 5 million deaths each year worldwide, making it the second leading cause of death and a major cause of disability [1]. This situation is becoming worse in the aged due to the increasing prevalence of some risk factors, like hypertension, coronary artery disease and atherosclerosis [2], [3]. Thrombolytic therapy is the only approved treatment for acute ischemic stroke [4], [5]. However, restoration of blood flow following thrombolytic treatment may aggravate the initial injury and lead to a second damage called I/R injury [6]. Neuroprotective approaches, as prospective newer treatments for stroke, have shown promise in animal models in recent decades, but their efficacies in patients remain limited [7]. Hence, there is an urgent need to develop effective neuroprotective agents to prevent and treat stroke.

Apoptosis has been suggested to be one of the main contributors to neuronal death in acute ischemic stroke [8], [9]. Strong evidence for neuronal apoptosis is seen in numerous I/R animal models [10], [11]. Moreover, apoptosis is evident in patients suffering from ischemic stroke [12], [13]. During stroke, a number of apoptosis regulatory gene products are activated [14], [15]. Over-expression of anti-apoptotic proteins such as Bcl-2 and Bcl-xL have been shown to promote cell survival after focal cerebral ischemia [16], [17], whereas Bcl-2 knockout exacerbated brain damage in stroke [18]. Furthermore, studies have demonstrated that Bcl-xL protein fusing to the protein transduction domain (PTD) of HIV TAT was protective in ischemia models [19], [20]. Therefore, targeted inhibition of apoptotic pathways may provide an attractive therapeutic approach for the treatment of ischemic brain injury.

14-3-3ε is a member of the 14-3-3 protein family, which bind to proteins and modulate interaction between proteins involved in various cellular functions such as intracellular signaling, cell cycling, apoptosis and transcriptional regulation [21], [22]. Proteomic analysis showed that 14-3-3ε expression is decreased approximately 50% in brain of a neonatal rat hypoxia/ischemia model [23], and the up-regulation of 14-3-3ε protein in brain protects cells against apoptosis [24]. 14-3-3ε is able to inhibit apoptosis by binding and sequestering phosphorylated Bad in the cytoplasm, thus preventing Bad translocation to mitochondria and interaction with Bcl-xL [24], [25]. Therefore, 14-3-3ε may function as an endogenous inhibitor of apoptosis, and its over-expression may be a promising strategy for treating ischemic stroke.

However, 14-3-3ε is unable to cross the BBB due to its large molecular weight of 30-kDa [21]. Protein transduction across BBB through PTDs is emerging as an attractive drug delivery strategy in various diseases or injuries [26]. The PTD sequence derived from HIV TAT protein is capable of delivering a large variety of proteins or peptides into the brain [27]. A number of TAT fusion proteins, such as TAT-Bcl-xL [19], [20], TAT-GDNF [28], Tat-Hsp70 [29], TAT-XIAP [30], TAT-neuroglobin [31], TAT-PARK7 [32], TAT-SOD [33] and TAT-NBD [34], [35], have been shown to efficiently cross the BBB after systemic administration and exhibit neuroprotective efficacy in cerebral ischemia animal models.

Autophagy, a cellular lysosome-mediated process, degrades and recycles subcellular organelles [36]. Increasing findings suggest that autophagy is activated following cerebral ischemia, but the contribution of autophagy to neuronal death/survival is still under debate [37], [38], [39], [40]. Recent studies have reported that 14-3-3ε is involved in the down-regulation of autophagy [41], [42]. However, little is known whether the regulation of autophagy by 14-3-3ε has protective or detrimental effects on ischemic brain injury.

Whether 14-3-3ε can be delivered into brain by TAT and TAT-14-3-3ε fusion protein has a protective effect against cerebral I/R injury is not known. In the present study, we prepared a TAT-14-3-3ε fusion protein, and evaluated its ability to cross BBB and transduce into the brain. We further examined the neuroprotective effect of TAT-14-3-3ε against rat transient focal cerebral ischemia. We demonstrated for the first time that systemic delivery of TAT-14-3-3ε protein results in a significant neuroprotection, indicating that TAT-14-3-3ε could be developed as a potential therapeutic agent in brain ischemic injury.

## Materials and Methods

### Ethics Statement

The experimental designs and all procedures were in accordance with the guidelines for the Care and Use of Laboratory Animals approved by Beijing Committee on Animal Care and Use. The protocol was approved by the Committee on the Ethics of Animal Experiments of the Peking University Health Science Center (Permit Number: LA2013-69). Efforts were made to reduce the number of animals used and their suffering.

### Codon Optimization and Cloning of Human 14-3-3ε Gene

The sequence encoding human *14-3-3ε* gene (GenBank accession number NR_024058) was generated by PCR. In order to facilitate the protein expression in *E*. *coli*, we modified the least preferred codons into the most preferred ones without changing amino acid sequence, according to the codon usage in *E. coli* [43]. A two-step strategy combining assembly PCR and overlap extension PCR process was used. A long codon-optimized DNA sequence was divided into three fragments with size from 200 to 350 bp, which was assembled via PCR reactions using 45 and 60 mer oligonucleotides (Table S1). There was a 15–20 bp overlap for each of the oligonucleotides used. These three fragments were assembled into a full-length DNA sequence by overlap extension PCR. The 768 bp fragments was ligated into the *pEASY*-T1 cloning vector (TransGen Biotech, Beijing, China) by TA cloning and confirmed by DNA sequencing.

### Construction of TAT–14-3-3ε Fusion Protein Expression Vectors

The 855 bp coding sequence of TAT-14-3-3ε fusion protein, which contains a 6×histidine tag leading sequence, followed by 11-amino-acid TAT-PTD and 14-3-3ε, was obtained by PCR. An oligonucleotide coding for a flexible spacer Gly-Gly-Ser was inserted to flank TAT 5′ and 3′, respectively. Restriction sites were added into the PCR primers to give the PCR products containing 5′ end *Nde*I and 3′ end *Bam*HI sites. The PCR product was subcloned into the *Nde*I-*Bam*HI digested pET-30a(+) plasmid (Novagen, Madison, WI, USA) resulting in recombinant expression vector pET-TAT-14-3-3ε. In addition, another plasmid pET-14-3-3ε coding for a 14-3-3ε control protein was constructed in a similar way. The insert in all the constructs was verified by DNA sequencing.

### Expression and Purification of TAT–14-3-3ε/14-3-3ε Fusion Proteins

The plasmid pET-TAT-14-3-3ε/pET-14-3-3ε was transformed into the *E. coli* BL21(DE3). A single clone was grown for 8 h at 37°C in 10 ml LB medium containing 50 μg/ml kanamycin. These cultures were diluted 50-fold with fresh LB media and cultured at 37°C to OD_600_ = 0.8. The expression of TAT-14-3-3ε and 14-3-3ε were induced by addition of 0.5 mM isopropyl-β-D-thiogalactoside (IPTG) for 10 h at 28°C. The bacterial cells were harvested by centrifugation at 10,000×g, and then resuspended in pre-cooled binding buffer (20 mM Tris–HCl, 500 mM NaCl and 20 mM imidazole, pH 7.5) and lysed by sonication on ice.

The supernatant containing target protein was collected by centrifugation 15,000×g for 20 min at 4°C and loaded onto a Ni^2+^-NTA resin column (Invitrogen) pre-equilibrated with binding buffer. The proteins were then eluted by a stepwise imidazole gradient in elution buffers (20 mM Na_3_PO_4_, 500 mM NaCl, 50–500 mM imidazole, pH 7.5). Eluted fractions were analyzed by 12% (wt/vol) SDS/PAGE, followed by Coomassie blue staining. Fractions that contained target protein were then pooled and dialyzed against water using a 5 kDa molecular weight cutoff dialysis membrane (Pierce, Rockford, IL, USA) at 4°C thoroughly. The purified proteins were lyophilized and kept at −80°C. The proteins were dissolved in normal saline (NS) and the protein concentrations were estimated by BCA protein assay (M&C Gene Technology, Beijing, China) before used, and the protein purity was also evaluated by SDS/PAGE. The purified recombinant proteins were confirmed by Western blot using anti-His-tag antibody (1∶1000, Abgent, CA, USA) and anti-14-3-3ε antibody (1∶3000, Abcam, Cambridge, UK).

### Transient Focal Cerebral Ischemia

Focal cerebral ischemia was induced by transient occlusion of the middle cerebral artery (MCAO) using the intraluminal filament technique as described previously [48]. Briefly, rats were anesthetized with chloral hydrate (350 mg/kg, i.p.), and the body temperature of rats was maintained at around 37°C with a heating pad and monitored by a rectal thermometer during surgery. The right common, external, and internal carotid arteries were exposed and common and external carotid arteries were permanently ligated with sutures. A 4-0 monofilament nylon suture with a rounded tip was introduced into the internal carotid artery through an incision in the common carotid artery and advanced 20–21 mm gently until the rounded tip reached the entrance to the right MCA. The monofilament was gently removed after 2 h to allow reperfusion of MCA, and the external carotid arteries was permanently ligated. Sham-operated rats were subjected to the same experimental procedures without the occlusion of MCA. After the surgery animals were returned to their home cages and closely monitored for 4–6 h.

### Animals and Experimental Groups

Adult male Sprague–Dawley rats (250–300 g, Department of Laboratory Animal Science of Peking University, Beijing, China) were given free access to food and water. Rats were randomly assigned to five groups: sham operated group, vehicle group (NS treated), 14-3-3ε treated group, and TAT-14-3-3ε treated group before or after ischemia (group I–V, respectively). Animals were sacrificed at 24 h of reperfusion after 2 h transient cerebral ischemia. Both 14-3-3ε and TAT-14-3-3ε were dissolved in NS. Intravenous injection of 0.3 ml NS (vehicle group), or 10 mg/kg 14-3-3ε (14-3-3ε treated group), or 10 mg/kg TAT-14-3-3ε (TAT-14-3-3ε pre-ischemic treated group) was performed 2 h before MCAO. In group V (post-ischemic treated group), 10 mg/kg TAT-14-3-3ε was intravenously injected at the end of ischemia.

In addition, the transduction of TAT-fusion protein across BBB often exhibits a significant increase at 4 h after administration [19], [28], [31], [44]. In order to corroborate the transduction of TAT-14-3-3ε, additional rats were injected intravenously with 10 mg/kg TAT-14-3-3ε or 14-3-3ε. At 4 h after injection, these animals were anesthetized and transcardially perfused with NS, and the cerebral hemispheres were quickly removed for Western blot analysis.

To determine whether the protective effects of TAT-14-3-3ε against transient focal cerebral ischemia is related to autophagy inhibition, rats were randomly assigned to the sham, vehicle, 3-MA and RAP + TAT-14-3-3ε groups. The treatment protocols and dosages of 3-MA and rapamycin were selected according to published studies [45], [46], [47]. The 3-MA (600 nmol, 5 μl, Sigma, M9281) or vehicle (normal saline, 5 μl) was administered using a single intracerebral ventricular (i.c.v.) injection at the onset of reperfusion. 3-MA was dissolved in normal saline by heating solution to 60–70°C and cooled to room temperature immediately before treatment. Rapamycin (35 pmol, 5 μl, i.c.v.) combined with TAT-14-3-3ε (10 mg/kg, i.v.) was delivered 2 h before MCAO, and then followed by 24 h reperfusion. Rapamycin (Sigma, R0395) was first dissolved in ethanol and then diluted in normal saline solution (the final ethanol concentration <2%).

### Western Blot Analysis for *In Vivo* Transduction

Transduction of the TAT-14-3-3ε fusion protein was verified using Western blot analysis. Proteins were extracted from the cerebral hemisphere in cold RIPA buffer (50 mM Tris, pH 7.4, 150 mM NaCl, 1% Triton X-100, 1% C_24_H_40_O_4_·Na, 0.1% SDS, 2 mM Na_4_P_2_O_7_, 25 mM β-glycerophosphate, 1 mM Na_3_VO_4_ and 1 mM PMSF) 4 h after intravenous injection of TAT-14-3-3ε or 14-3-3ε. Equal amounts of protein (40 μg) were diluted in 6×sample buffer, boiled and separated by 12% (wt/vol) SDS/PAGE, and then blotted onto PVDF membrane (Millipore, Billerica, MA, USA) using a wet transfer system (Bio-Rad, Hercules, CA, USA). The membrane was then immersed in blocking solution [5% milk in TBST (0.1% Tween 20 + TBS)] for 2 h at room temperature, followed incubated with a monoclonal rabbit anti-14-3-3ε antibody (1∶1000, Abcam, Cambridge, UK), rabbit anti-His-tag antibody (1∶1000, Abgent, CA, USA) or rabbit anti-β-actin antibody (1∶2000, Santa Cruz Biotechnology, CA, USA) overnight at 4°C. Subsequently, membranes were incubated with a horseradish peroxidase-labeled goat anti-rabbit secondary antibody (1∶5000, Santa Cruz Biotechnology, CA, USA), followed by ECL detection (Pierce, Rockford, IL, USA). The density of each band was quantified using the Quantity One software.

### Evaluation of Neurological Deficit

The animals were subjected to a neurological examination after 24 h reperfusion using an established scoring system [49], and the observer was blinded to the identity of the treatment groups. Neurological deficit was scored on a 5-point scale (0, no neurological deficit; 1, failure to extend right paw fully; 2, circling to right; 3, falling to right; 4, unable to walk spontaneously and having depressed levels of consciousness).

### Infarct Volume Analysis

After scoring neurological function, the animals were anesthetized with chloral hydrate (350 mg/kg, i.p.) and transcardially perfused with NS. Brains were collected rapidly and sectioned into five 2-mm-thick coronal slices, and stained with 1% 2,3,5-triphenyltetrazoliumchloride (TTC) (Amresco, Solon, CA, USA) at 37°C for 30 minutes. The slices were fixed in 4% formalin overnight and then photographed with a digital camera (PowerShot G12, Canon). Unstained areas (pale color) were defined as ischemic lesions. The areas of the infarction were analyzed using Image J software (NIH Image), by measuring the surface of each slice and numerically integrated across the thickness of the slice to obtain the infarct volume. Volumes from all five slices were summed to calculate total infarct volume over the entire hemisphere, expressed as mm^3^. To compensate for the effect of brain edema, the corrected infarct volume was calculated as follows: the corrected infarct volume  =  volume of contralateral hemisphere – (volume of ipsilateral hemisphere – volume of infarct) [50].

### TUNEL staining

Rats were anesthetized deeply with chloral hydrate (350 mg/kg, i.p.) and transcardially perfused with 4% paraformaldehyde (n = 3). Then brains were removed and fixed in 10% buffered formalin solution for 48 h, and then embedded in paraffin. Terminal deoxynucleotidyl transferase-mediated dUTP nick end-labeling (TUNEL) staining was performed to investigate the apoptosis according to the protocol of *in situ* apoptosis detection kit (KeyGen Biotech, Nanjing, China). Brains were cut into 10 μm thick coronal sections at the level of the bregma, and then incubated with a TUNEL mixture containing 0.5 U/μl TDT, 0.2 nmol biotin-11-dUTP, followed by streptavidin-FITC. TUNEL staining was detected under a fluorescence microscope (Olympus IX71). Three sections from each animal were viewed by two observers blinded to the origin of the slides. For each section, the TUNEL positive cells were counted in 10 non-overlapping high-power fields (×200) covering the majority of striatum.

### Caspase-3 Activity Assay

The activity of caspase-3 was detected using a Caspase-3 Colorimetric Assay Kit (KeyGen Biotech, Nanjing, China) following the manufacturer's instruction. Proteins were extracted from fresh right cortical samples 24 h after MCAO (n = 4). Tissues were homogenized in cold lysis buffer containing 25 mM HEPES, pH 7.5, 5 mM MgCl_2_, 2 mM EDTA, 0.1% Triton 100, 2 mM dithiothreitol (DTT) and 1 mM PMSF. The homogenates were centrifuged at 13,000 ×g for 30 min at 4°C, and the supernatants were collected. The protein concentration was determined by BCA assay (M&C Gene Technology, Beijing, China) and adjusted to 2 μg/μl. Then 100 μg proteins were added to 50 μl 2×Reaction Buffer supplement with 5 μl Caspase-3 Substrate and then incubated at 37°C away from light for 4 h. Caspases-3 activities were quantified spectrophotometrically at a wave length of 405 nm in a microplate reader (Thermo Scientific, Waltham, MA, USA). The activity of caspase-3 was expressed as the fold increase over the sham group.

### Determination of Neuronal Viability

To evaluate the degree of ischemic neuronal damage, Nissl staining was performed according to classical methods. The brain sections described above were stained with cresyl violet (Beyotime Institute of Biotechnology, Haimen, China). Cells that contained Nissl substance were considered to be viable neurons. Three sections from each animal (n = 4) were selected for analysis. The viable neurons were quantified by manually counting cells with visible nucleoli in 10 randomly chosen, non-overlapping high power fields (×200) of striatum using microscopy (Olympus IX71) in a blinded fashion.

### Determination of I/R-induced Autophagy by Western Blot Analysis and Transmission Electron Microscopy (TEM)

For autophagy analysis, rats were subjected to 2 h MCAO followed by 24 h reperfusion, and then proteins were extracted from the ischemic cerebral hemisphere and detected by Western blot analysis. Polyclonal rabbit anti-LC3B antibody (1∶1000, Santa Cruz, CA, USA), polyclonal rabbit anti-Beclin-1 antibody (1∶1000, Santa Cruz, CA, USA) and polyclonal rabbit anti-p62/SQSTSM1 antibody (1∶500, Abgent, CA, USA) were used for immunoreactivity detection.

TEM was used to evaluate the ultrastructural changes. After 24 h of reperfusion, rats were deeply anesthetized and sacrificed by transcardial perfusion with 50 ml NS followed by 50 ml of fixative (2% glutaraldehyde and 2% paraformaldehyde). Next, the cerebral fragments were fixed with 2.5% glutaraldehyde solution overnight at 4 °C, followed by immersion in 1% osmium tetroxide in 0.1 mol/l phosphate buffer (pH 7.4) for 2 h. The tissues were dehydrated in ascending series of ethanol before embedding samples in araldite. The ultrathin sections were cut and doubly stained with uranyl acetate and lead citrate, and examined with a JEM-1400 type transmission electron microscope (Electron Co, Japan) and photographed.

### Statistical Analysis

All values are expressed as means ± SD. The one way ANOVA was used to test for differences among groups, followed by post hoc Scheffe's tests. P<0.05 was considered statistically significant.

## Results

### Generation of Pure TAT-14-3-3ε and 14-3-3ε Fusion Proteins

**Figure 1:**
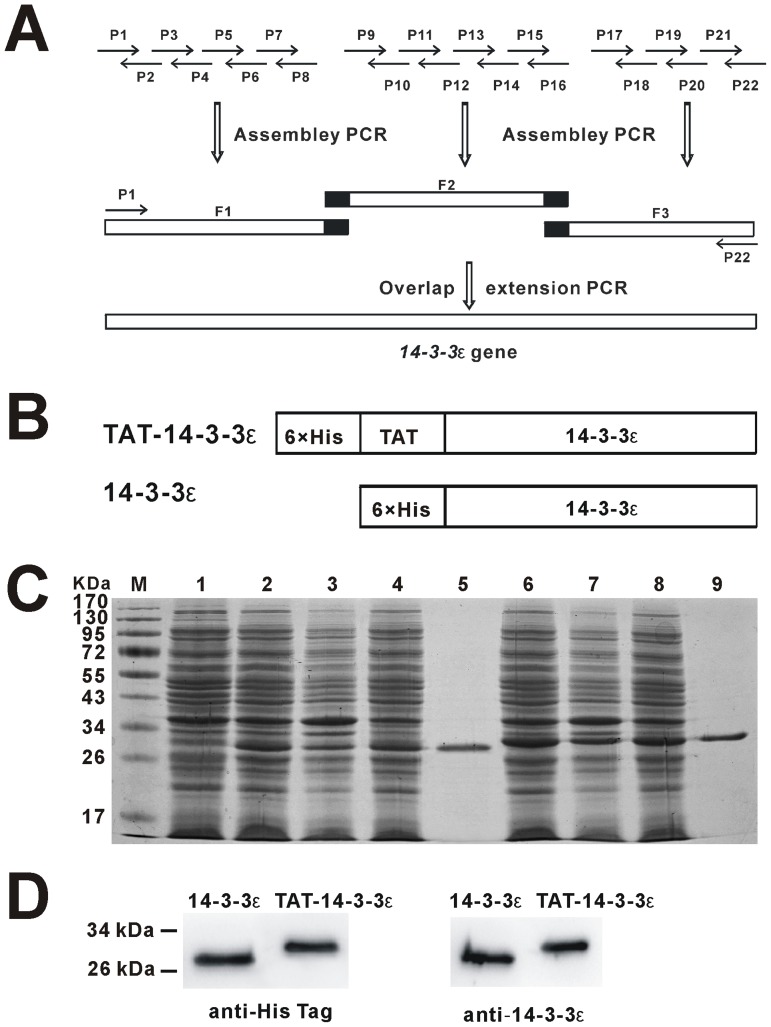
Generation of the fusion proteins TAT-14-3-3ε and 14-3-3ε. (A) The strategy for the synthesis and assembly of the *14-3-3ε* gene using two step PCR. (B) Diagram of TAT-14-3-3ε and control 14-3-3ε fusion proteins. (C) Expression and purification of TAT-14-3-3ε and 14-3-3ε proteins from bacteria. *E. coli* BL21(DE3) cells with TAT-14-3-3ε or 14-3-3ε plasmid were induced as described in Materials and Methods. Protein samples were separated by SDS/PAGE and the protein bands are shown after Coomassie blue staining. Lane M, prestained protein markers; Lane 1, total cell proteins before induction; Lane 2-4, whole cell lysate, inclusion body and cellular lysate supernatant of induced BL21 (DE3)/pET-14-3-3ε induced at 28°C, respectively; Lane 5, Purified His-tagged 14-3-3ε proteins. Lane 6-8, whole cell lysate, inclusion body and cellular lysate supernatant of induced BL21 (DE3)/pET-TAT-14-3-3ε induced at 28°C, respectively; Lane 9, Purified His-tagged TAT-14-3-3ε proteins. (D) Western blot analysis of purified His-tagged 14-3-3ε and TAT-14-3-3ε with anti-His-tag antibody and anti-14-3-3ε antibody.

Codon-optimized 14-3-3ε DNA sequence was generated by a two-step PCR strategy as shown in Figure 1A, and has been deposited in the GeneBank (accession number KC733246). We constructed TAT-14-3-3ε fusion protein, in which 14-3-3ε was fused in frame with TAT-PTD domain and 6×His-tag, and 14-3-3ε fused to His-tag alone as a negative control (Figure 1B). Both protein-coding sequences were cloned into pET-30a(+) vector for protein expression. The recombinant fusion proteins were expressed mainly in soluble form when induced by IPTG at 28°C (Figure 1C). After purification by Ni-NTA affinity chromatography, only one single band appeared for both TAT-14-3-3ε and 14-3-3ε protein, as determined by Coomassie brilliant blue stained SDS/PAGE (Figure 1C). Recombinant TAT-14-3-3ε and 14-3-3ε proteins showed a molecular mass of approximately 31 kDa and 29 kDa, respectively. The purified fusion proteins were further corroborated by Western blot analysis with anti-His-tag and anti-14-3-3ε antibodies (Figure 1D).

### Efficient Transduction of TAT-14-3-3ε Fusion Protein into Brain *In Vivo*

**Figure 2:**
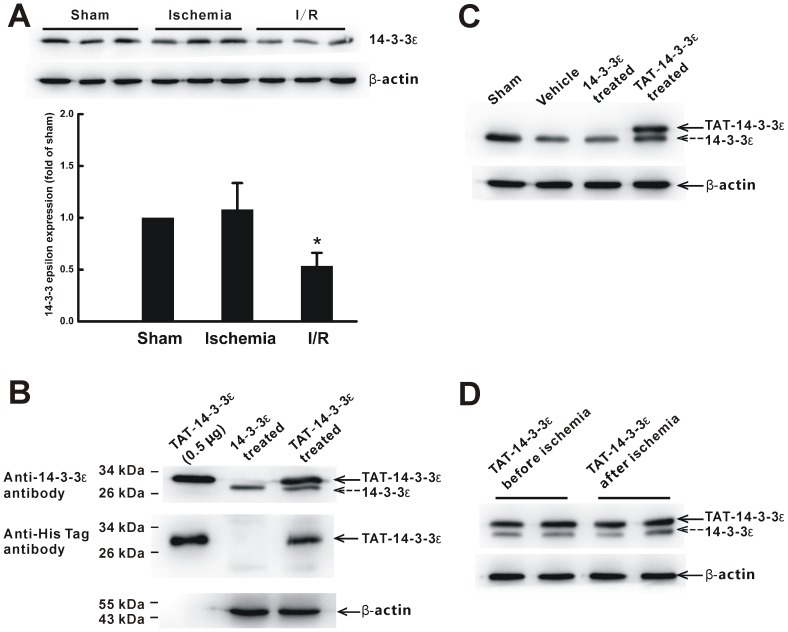
*In vivo* protein transduction of TAT-14-3-3ε in rat brain. (A) 14-3-3ε protein levels in brain tissues of sham, 2 h ischemia or I/R group. I/R, 2 h ischemia followed by 24 h reperfusion. Lower panel shows the quantification of 14-3-3ε levels in upper panel. Each bar denotes mean ± SD. *p<0.05 compared with sham group. (B) Representative Western blot analysis of TAT-14-3-3ε transduction in the rat brain after intravenous infusion of protein using anti-14-3-3ε or anti-His-tag antibody. In the TAT-14-3-3ε treated rat brain, both the TAT-14-3-3ε proteins (as indicated by the solid arrow) and 14-3-3ε proteins (as indicated by the dashed arrow) were detectable in the brain. In the 14-3-3ε-treated rats, only the 14-3-3ε proteins were detectable in the brain (n = 3 per group). Purified TAT-14-3-3ε fusion protein was provided as a positive control. (C) Representative Western blotting with anti-14-3-3ε antibody shows the 14-3-3ε protein levels (including endogenous 14-3-3ε and exogenous TAT-14-3-3ε proteins) in the brains of rats treated with vehicle, 14-3-3ε, TAT-14-3-3ε 2 h before ischemia, 4 h after intravenous administration. Similar results were obtained in 2 other experiments. (D) Western blotting with the anti-14-3-3ε antibody detects the transduction of TAT-14-3-3ε into brain 4 h after intravenous injection, either before ischemia or after ischemia. In all blots, β-actin was used as an internal loading control. Each lane represents an individual animal. The blots are representative of two independent experiments with similar results.

Western blot analysis using anti-14-3-3ε antibody showed that the expression levels of endogenous 14-3-3ε protein in brain tissues of I/R rats were decreased by half (Figure 2A), confirming previous report [23]. To detect whether the TAT-14-3-3ε could pass BBB, Western blot analysis was performed using anti-14-3-3ε and anti-His-tag antibodies. At 4 h after intravenous injection of TAT-14-3-3ε or 14-3-3ε (both His-tagged), the brain was thoroughly flushed with NS by perfusion to exclude the potential effect of recombinant proteins in the blood. Robust His-tag immunoreactivity was detected in the brain lysate after intravenous injection of TAT-14-3-3ε, and 14-3-3ε treated animals did not show specific His-tag immunoreactivity (Figure 2B). In TAT-14-3-3ε-treated animals, two visible bands were detected in the brain using anti-14-3-3ε antibody, and the upper robust band exhibited virtually identical to TAT-14-3-3ε, with a molecular mass of 31 kDa, and the lower band was endogenous 14-3-3ε protein. Moreover, quantitative analysis of the relative intensities of each band indicated that the exogenous TAT-14-3-3ε level was about 3 times higher than the endogenous 14-3-3ε protein. However, in 14-3-3ε-treated control animals, only the endogenous 14-3-3ε protein was detected. The results suggest that TAT-14-3-3ε efficiently crosses intact BBB and transduces into brain after administrated intravenously and wild-type 14-3-3ε without TAT cannot cross BBB. Next, the levels of total 14-3-3ε protein in the ischemic brain were examined by Western blotting, and the results showed that the levels of exogenous 14-3-3ε protein were increased in TAT-14-3-3ε treated rats (Figure 2C). We further confirmed that there was no significant difference of the transduction of TAT-14-3-3ε in the brain between TAT-14-3-3ε pre-treated 2 h before ischemia and post-ischemic treatment at the end of ischemia (Figure 2D).

### Protection of TAT-14-3-3ε Fusion Protein against Focal Ischemic Injury

**Figure 3:**
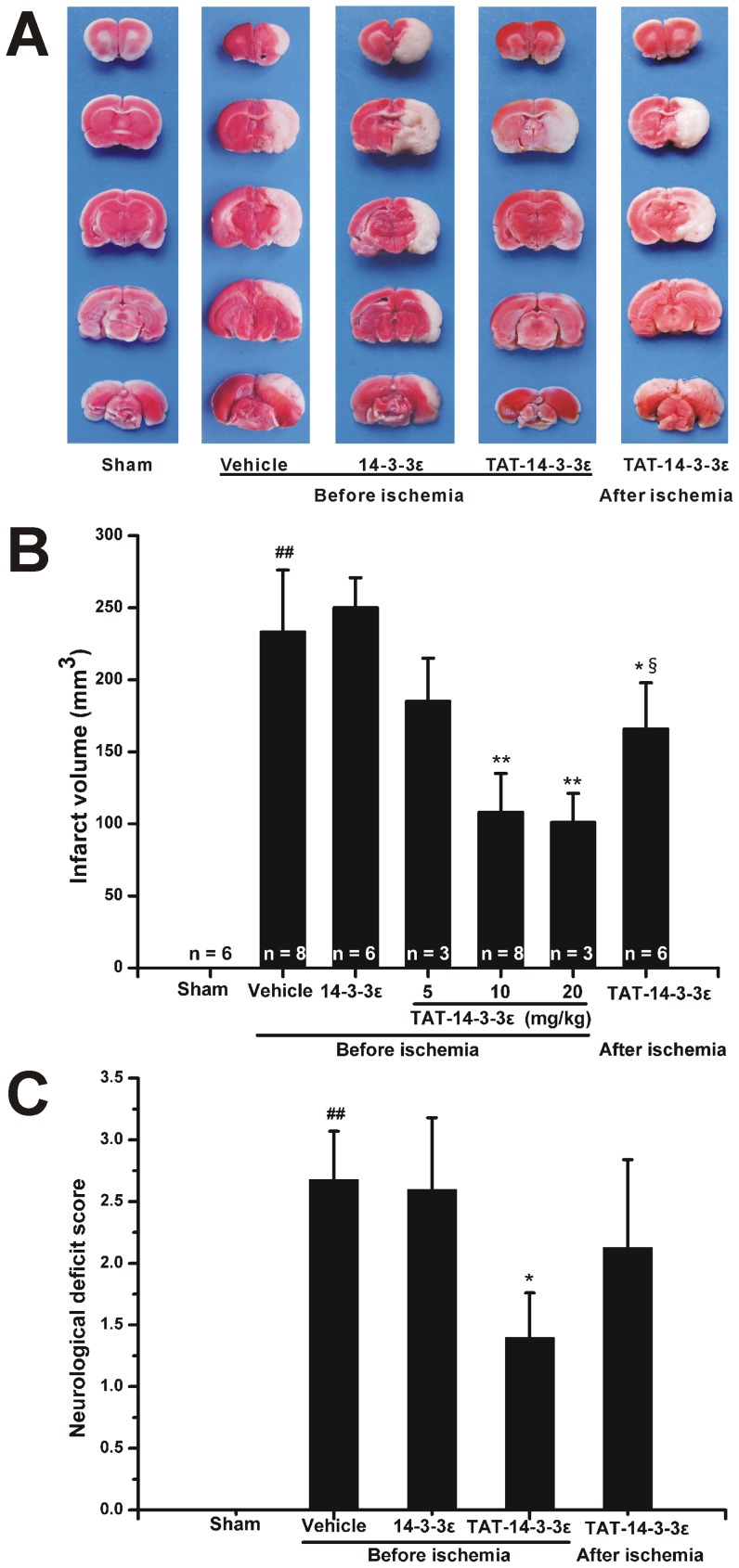
Protection of TAT-14-3-3ε on infarct volumes and neurological deficit scores of rats subjected to 2 h MCAO followed by 24 h reperfusion. (A) Representative TTC-stained brain coronal sections of rats treated with vehicle, 14-3-3ε, TAT-14-3-3ε 2 h before ischemia and TAT-14-3-3ε at the end of ischemia. Cerebral infarct volumes (B) and neurological deficit scores (C) of rats treated with vehicle, 14-3-3ε, TAT-14-3-3ε 2 h before ischemia or at the end of ischemia. Note that TAT-14-3-3ε treatment significantly reduced infarct volume and ameliorated neurological performance. ##*p*<0.01 compared with sham group. *p<0.05 and **p<0.01 compared with vehicle and 14-3-3ε treated group. §*p*<0.05 compared with TAT-14-3-3ε (10 and 20 mg/kg) pre-treated group.

**Figure 4:**
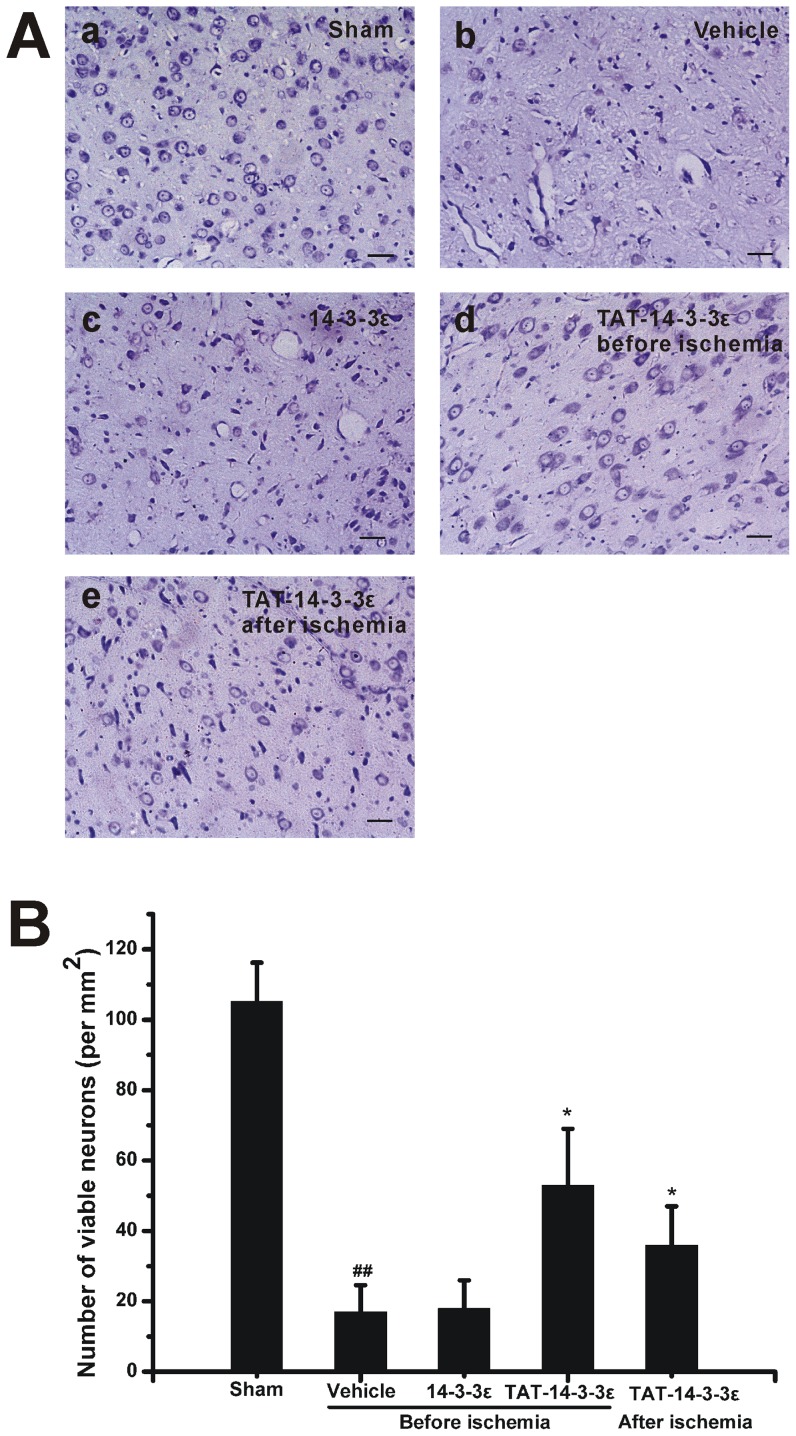
Nissl staining for coronal brain sections of rats subjected to 2 h of MCAO and 24 h of reperfusion. (A) Representative photographs of cresyl violet-stained cells in the striatum. Rats were subjected to sham operation (a) and rats subjected to 2 h of ischemia followed by 24 h of reperfusion with administration of vehicle (b), 14-3-3ε (c), TAT-14-3-3ε 2 h before ischemia (d), TAT-14-3-3ε at the end of ischemia (e). Data were obtained from four independent animals and the results of a typical experiment are presented. (B) Quantification of the viable nerve cells shows that TAT-14-3-3ε significantly increased neuronal viability compared with vehicle and 14-3-3ε treated animals. The data are expressed as the number of surviving neurons per mm^2^ in the striatum counted under light microscopy. Data are mean ± SD (n = 4 per group). ##*p*<0.01 compared with sham group. *p<0.05 compared with vehicle and 14-3-3ε treated group. Scale bar  = 50 μm.

To determine whether the increased 14-3-3ε protein levels in the brain can protect neurons against ischemic damage, we examined infarct volume and neurological deficits after treatment with recombinant 14-3-3ε proteins in rat focal cerebral I/R model. We first detected the function of TAT-14-3-3ε at three doses (5, 10 and 20 mg/kg) (Figure 3B). A significant reduction of infarct volume was reached at 10 mg/kg, and less reduction at 5 mg/kg. Additional increase in the dose to 20 mg/kg did not result in an additional significant protection against the focal ischemic damage. Thus the following experiments were performed at a dose of 10 mg/kg. The animals treated with vehicle or control 14-3-3ε protein developed severe infarct involving the cerebral cortex and striatum, and the brain infarct volumes and neurological deficits showed no significant difference between them (Figure 3). In contrast, pre-treatment with TAT-14-3-3ε 2 h before ischemia significantly reduced infarct volumes (Figure 3A and B). In addition, post-ischemic treatment of the animals with TAT-14-3-3ε at the end of ischemia reduced brain infarct volume, but the protective effect appeared not as strong as that in pre-treatment. Similarly, animals pre-treated with TAT-14-3-3ε showed a significant improvement in neurological functions (Figure 3C), indicating a consistent and significant correlation with the infarct size. There was also a trend towards a reduction of neurological deficits in TAT-14-3-3ε post-treatment group. We examined the neuronal survival by cresyl violet (Nissl) staining. Accordingly, the number of surviving neurons in the striatum was significantly increased in either TAT-14-3-3ε treatment groups versus vehicle or control 14-3-3ε groups (Figure 4A and B). Taken together, pre-treatment with TAT-14-3-3ε significantly protects against focal cerebral ischemic injury and improves neuronal survival.

### Effect of TAT-14-3-3ε on Neuronal Apoptosis in Ischemic Brain

**Figure 5:**
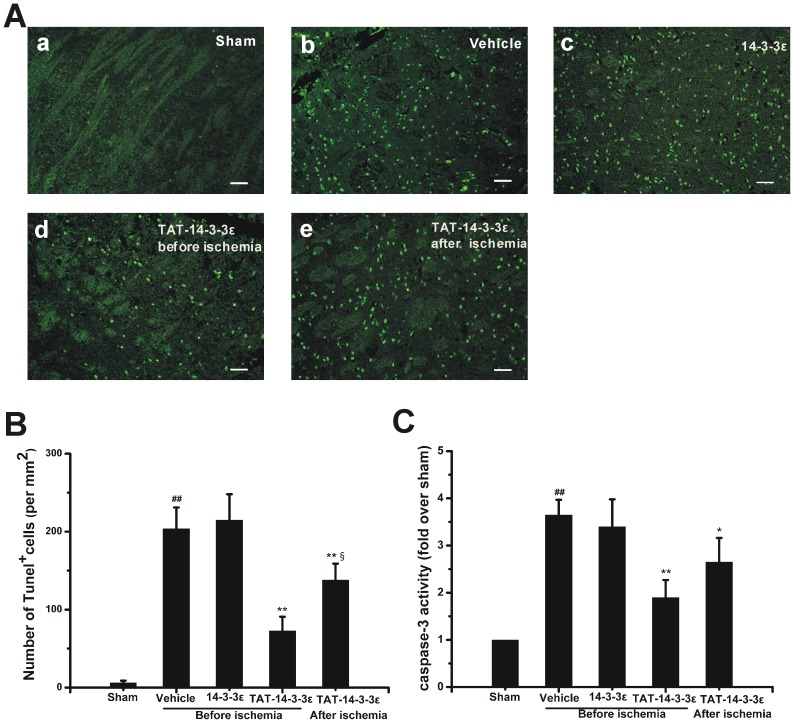
TAT-14-3-3ε reduces ischemia-induced neuronal cell apoptosis and caspase-3 activation. Rats were subjected to MCAO for 2-3-3ε, TAT-14-3-3ε 2 h before ischemia or TAT-14-3-3ε at the end of ischemia, respectively. (A) Representative photographs shows TUNEL staining cells in the striatum of rats. (B) Quantification of the densities of TUNEL-positive cells shown in *A* (n = 3 per group). Note that TAT-14-3-3ε significantly reduces the density. (C) Colorimetric detection of caspase-3 inhibition by TAT-14-3-3ε. Values given are mean ± SD (n = 4 per group). ##*p*<0.01 compared with sham group. *p<0.05 and **p<0.01 compared with vehicle and 14-3-3ε treated group. §*p*<0.05 compared with TAT-14-3-3ε pre-treated group. Scale bar  = 50 μm.

To explore the cellular mechanisms underlying the neuroprotective efficacy of TAT-14-3-3ε, we examined the apoptotic death by using TUNEL staining and caspase-3 activity colorimetric assay. The number of apoptotic cells in the striatum zone was significantly reduced in both pre-treatment and post-ischemic treatment groups with TAT-14-3-3ε, as compared with vehicle or control 14-3-3ε protein treated group (Figure 5A and B). The caspase-3 activity in both TAT-14-3-3ε treated groups was also found to be significantly decreased, as compared with that in vehicle or control 14-3-3ε treated animals. Besides, both the apoptotic cells and caspase-3 activity in TAT-14-3-3ε pre-treatment animals were significantly less than that in TAT-14-3-3ε post-treatment group, implying that pre-ischemic treatment with TAT-14-3-3ε more effectively protects against neuronal apoptosis before it occurs. These data collectively demonstrate that TAT-14-3-3ε attenuates I/R-induced neuronal apoptosis.

### Effect of TAT-14-3-3ε on Autophagy in Cerebral Ischemia

**Figure 6:**
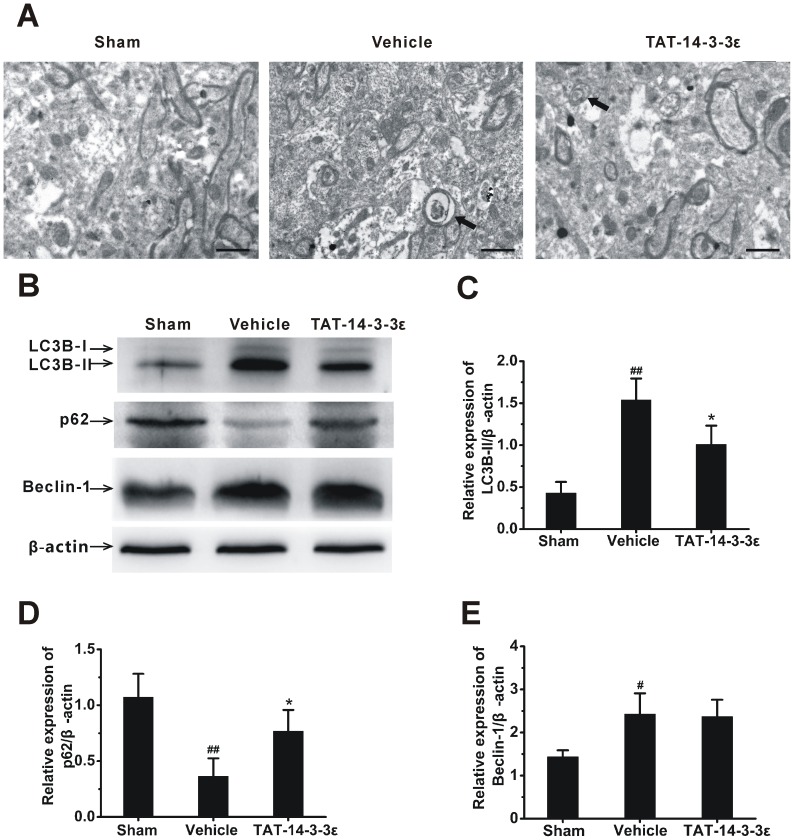
TAT-14-3-3ε inhibits I/R-induced autophagy in brain. (A) Representative electron microphotographs showing autophagosomes in the ischemic penumbra of cerebral cortex of sham, vehicle or TAT-14-3-3ε pre-treated animals 24 h after reperfusion. Autophagosomes are indicated by arrows. Scale bar  = 1 μm. (B) Western blot analysis for the expression of LC3B, p62 and Beclin-1 in cerebral hemisphere. (C, D and E) Quantitation of LC3B-II, p62 and Beclin-1 expression, respectively, normalized to β-actin. The data are presented as the mean ± SD (n = 5 per group). #*p*<0.05 and ##*p*<0.01 versus sham group. *p<0.05 versus vehicle group.

To further delineate the neuroprotective mechanisms of TAT-14-3-3ε in ischemic brain damage, we examined the effect of TAT-14-3-3ε on I/R-induced autophagy by TEM analysis and Western blot analysis with autophagy specific antibodies against microtubule-associated protein 1A light chain 3 (LC3), autophage-related gene 6 (Atg-6)/Beclin-1 and p62/SQSTM1. Ultrastructural analysis (Figure 6A) with TEM showed that neuronal morphology appeared to be normal in the sham group. At 24 h after I/R, cell shrinkage, loss of cellular organelles and formation of autophagosomes were observed in the ischemic penumbra of cerebral cortex. However, most of the neurons and their organelles appeared to be normal in the TAT-14-3-3ε treated group, with a less neuronal damage. In addition, autophagosomes were less frequently observed than that in I/R groups. Concurrent increase of both LC3 and Beclin-1 represents activation of autophagy [51]. The protein expression levels of the membrane-bound form LC3B-II were significantly increased in the ischemic cerebral hemisphere 24 h after I/R as compared with sham control group. Pretreatment with TAT-14-3-3ε significantly but partially blocked ischemic injury-induced up-regulation of LC3B-II (Figure 6B and C). The protein levels of Beclin-1, a positive regulator of autophagy [51], [52], were significantly increased at 24 h after ischemic brain injury. Pretreatment with TAT-14-3-3ε did not produce any significant effect on ischemia-induced up-regulation of Beclin-1 expression (Figure 6B and E). Accordingly, treatment with TAT-14-3-3ε increased the levels of p62 (Figure 6B and D), a protein selectively degraded by autophagy [53]. These results indicated that increased 14-3-3ε protein in the brain inhibits I/R-induced autophagy.

**Figure 7:**
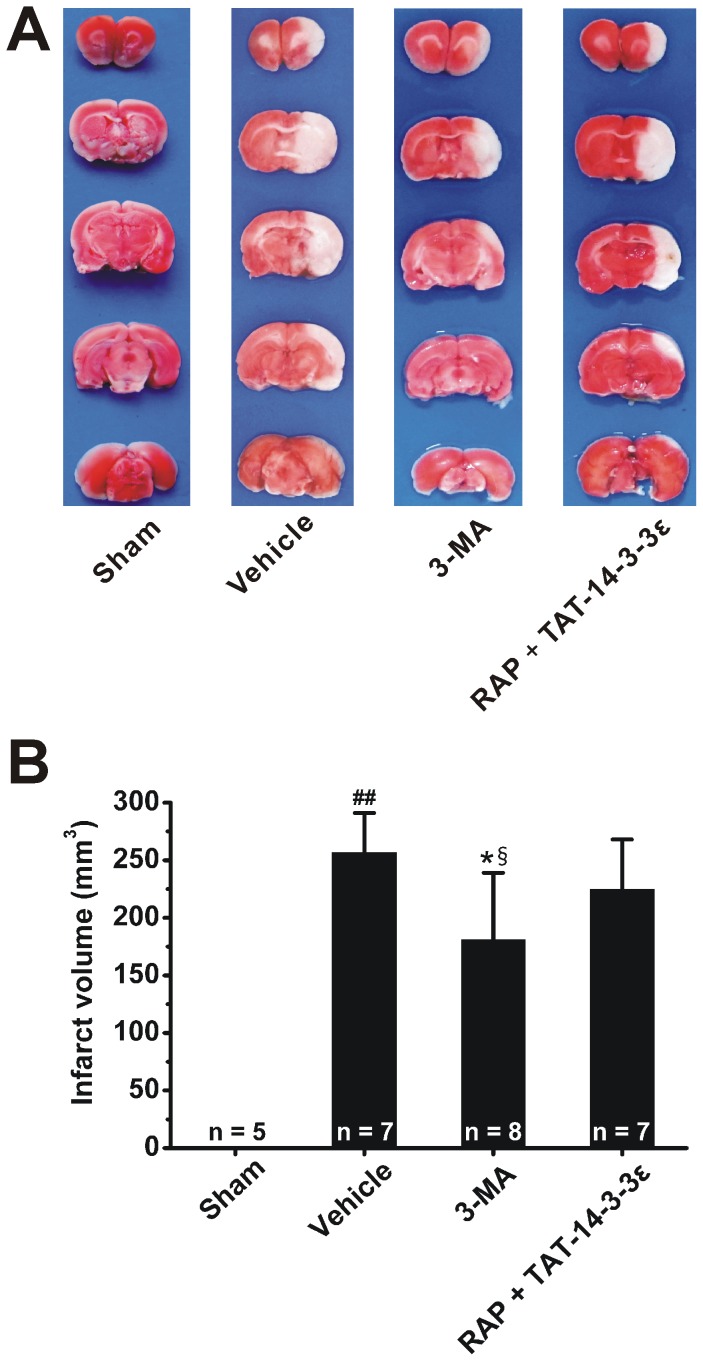
Rapamycin pretreatment attenuated the neuroprotective effects of TAT-14-3-3ε. Rats were treated with a single i.c.v. injection of saline or 600-MA at the onset of reperfusion, or RAP (35 pmol, i.c.v.) combined with TAT-14-3-3ε (10 mg/kg, i.v.) 2 h before ischemia, and then followed by 24 h reperfusion. (A) Representative TTC staining from rat brains in the sham, vehicle, 3-MA and RAP + TAT-14-3-3ε groups. (B) Quantitative analysis of brain infarct volume from each group. Note that 3-MA decreases the infarct volume, and RAP weakens the neuroprotective effects of TAT-14-3-3ε. Bar represents mean ± SD, ##*p*<0.01 compared with sham group. *p<0.05 compared with vehicle group. §*p*<0.05 compared with RAP + TAT-14-3-3ε group.

**Figure 8:**
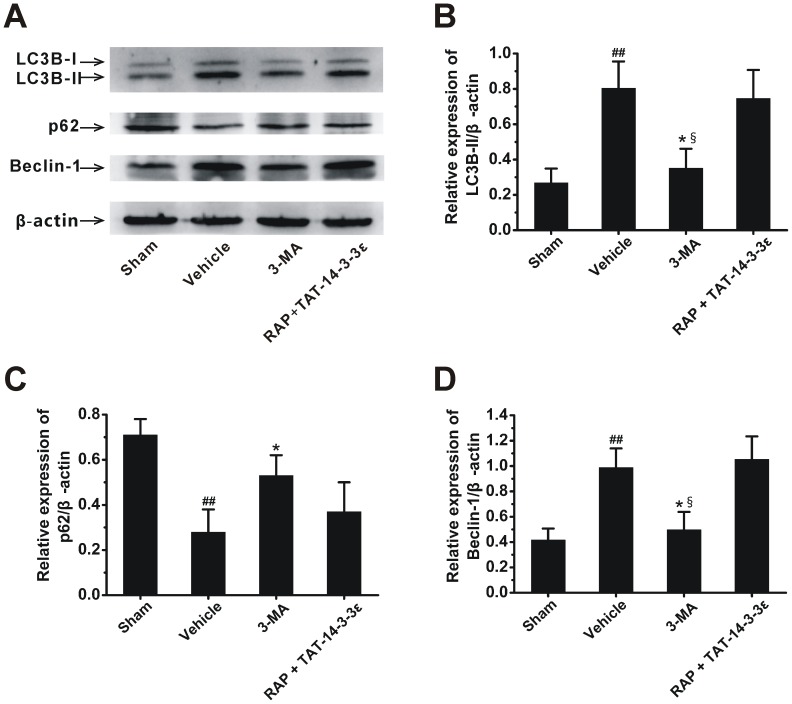
Inhibition of autophagy contributes to the neuroprotective effects of TAT-14-3-3ε. Rats were treated with an i.c.v of saline or 3-MA (600 nmol) at the end of 2h ischemia, or RAP (35 pmol, i.c.v.) combined with TAT-14-3-3ε (10 mg/kg, i.v.) 2 h before 2 h MCAO. Rats were then subjected to 24 h reperfusion, after which all animals were sacrificed. (A) Western blot analysis of the LC3B, p62 and Beclin-1 protein expression in the ischemic cerebral hemisphere. (B, C and D) Quantitation of LC3B-II, p62 and Beclin-1 expression, respectively, normalized to the loading control (β-actin). Bar represents mean ± SD (n = 4 per group). ##*p*<0.01 compared with sham group. *p<0.05 compared with vehicle group. §*p*<0.05 compared with RAP + TAT-14-3-3ε group.

We further tested whether the regulation of TAT-14-3-3ε on autophagy contributed to the neuroprotective effect of TAT-14-3-3ε in ischemic stroke. First, we determined the effect of autophagy on ischemic injury in a rat model using autophagy inhibitor 3-MA. Rats were subjected to 2 h MCAO followed by 24 h reperfusion. The result showed that i.c.v. injection of 600 nmol 3-MA at the onset of reperfusion reduce the infarct size. In contrast, combined treatment (2 h before MCAO) of TAT-14-3-3ε and autophagy inducer rapamycin (35 pmol, i.c.v.) partly attenuated the protective effects of TAT-14-3-3ε on infarct volume (Figure 7 and 3). Western blot analysis revealed that the protein levels of LC3B-II and Beclin-1 in ischemic cerebrum were significantly down-regulated (Figure 8A, B and D) and p62 expression was up-regulated in the 3-MA group (Figure 8A and C), which confirmed that 3-MA block the autophagy activation in cerebral I/R [45], [46], [47]. However, there was no significant difference in the expression of LC3B-II, Beclin-1 and p62 among the vehicle and RAP + TAT-14-3-3ε groups, suggesting that the inhibition of autophagy activation in ischemic brain mediated by pretreatment with TAT-14-3-3ε was partly reversed by autophagy inducer rapamycin (Figure 8). The present study further demonstrates that the neuroprotection induced by TAT-14-3-3ε pretreatment, at least partly, mediated by inhibition of autophagy activation in a rat I/R model.

## Discussion

The salient finding in this study is the significant neuroprotection of TAT-14-3-3ε against focal cerebral ischemia. We prepared a novel TAT-14-3-3ε fusion protein using codon-optimization and recombinant protein expression/purification technology. TAT-14-3-3ε readily crossed BBB and effectively transduced brain following intravenous injection even under intact BBB conditions. Pre- or post-ischemic treatment with TAT-14-3-3ε significantly reduced cerebral infarct size and improved neurologic outcomes in a rat focal cerebral I/R model. Such neuroprotection is attributed to the improved neuronal survival due to the significant reduction of neuronal apoptosis mediated by caspase-3 activity, and the inhibition of autophagy. These data confirm the neuroprotective effect of 14-3-3ε via inhibiting neuronal apoptosis *in vivo* [24], [54], and provide first evidence that engineered TAT-14-3-3ε fusion protein could be developed as a novel therapeutic agent for the treatment of stroke and possibly other neurodegenerative diseases.

TAT-mediated transduction is able to cargo large molecules such as proteins across cell membranes, and is becoming a promising tool for the therapy of many diseases [27], [55]. In ischemic stroke, therapeutic drugs are required to enter the central nervous system. Thus TAT-mediated delivery is of particular significance, due to its high efficiency in crossing intact BBB [56], [57], [58] and easy transduction in cells. In this study, the fusion protein contains a His-tag, which allows immunodetection of the transduced protein to be distinguished from endogenous 14-3-3ε. The exogenous 14-3-3ε fused to TAT-PTD was delivered efficiently into the intact brain when administrated peripherally, as confirmed by Western blot analysis showing 3-fold increase of 14-3-3ε in brain at 4 h after intravenous injection. The efficient transduction of TAT-14-3-3ε fusion protein supports previous reports showing that transduction of TAT-Bcl-xL [19] and TAT-Neuroglobin [31] into the intact brain peaks at 4 h after administration. Therefore, TAT-14-3-3ε holds high potential for clinical applications. Figuring out the target cells of TAT-14-3-3ε can be very meaningful. TAT conjugated proteins can enter primary neurons, astrocytes and cultured neuronal cell lines *in vitro*, such as TAT-Bcl-2△loop [59], TAT-Hsp70 [29] and TAT-DJ-1[60]. Others have observed that TAT-Bcl-xL [19] and TAT-XIAP [30] were detected both in a large number of neurons and some astrocytes *in vivo*. Besides, there were still many TAT fusion proteins showing effect in animal model, but the cellular distribution of these proteins was not yet analyzed, such as TAT-GDNF [28] and TAT-NEP1-40 [61]. As TAT proteins enter various cell types in a non-selective manner with high efficiency [62], we speculate that TAT-14-3-3ε protein may be transduced into all cells in brains, including neurons, astrocytes, oligodendrocytes, microglia/macrophages, neural stem/progenitor cells, endothelial cells and ependymal cells.

Consistent with previous reports [23], [24], there is a half reduction in 14-3-3ε expression after ischemic brain injury in the present animal models, and the increase of 14-3-3ε in brain significantly protects against ischemic damage [24]. So it would be attractive and beneficial to rapidly achieve a high and therapeutic level of 14-3-3ε in the ischemic brain. The present results demonstrated that pre-ischemic administration of TAT-14-3-3ε significantly reduces the brain ischemic infarct volume, improves neurologic scores and increases neuronal survival, suggesting the transduction of TAT-14-3-3ε provides a strong neuroprotective effect. In addition, post-ischemic treatment with TAT-14-3-3ε also exhibits a protection against brain ischemia, although it is less effective. As there is no significant difference in the transduction of TAT-14-3-3ε in the brain under ischemic and non-ischemic conditions, the data suggest that less effective of post-ischemic TAT-14-3-3ε treatment is not due to the possibility of lower levels of delivered TAT-14-3-3ε in the injured brain tissues. Neurons are highly sensitive to ischemia and hypoxia injury, so the earlier intervention in the pathological process begins, the better therapeutic effect is obtained. The inferior effectiveness of TAT-14-3-3ε post-ischemia treatment is most probably due to ongoing loss of Neurons. However, the underlying mechanisms need further investigation.

Accumulating evidences suggest that 14-3-3ε displays important anti-apoptotic characteristics [21], [63], [64]. Our results showed that TAT-14-3-3ε reduced the TUNEL-positive cells significantly, implying that TAT-14-3-3ε could prevent the apoptotic modes of neuronal cell death in rats. Previous studies have demonstrated that 14-3-3ε binds to p-Bad, resulting in retention of p-Bad in the cytoplasm and preventing Bad from entering mitochondria, and thus prevent the subsequent mitochondria-mediated apoptosis [21], [24], [25]. Caspase-3 is an important and common effector in the process of apoptosis, and its inhibition can block apoptotic cell death [65]. Our study proves that intravenous administration of TAT-14-3-3ε decreased caspase-3 activity in brain I/R, by both pre-ischemic and post-ischemic treatments. Taken together, these results indicate that TAT-14-3-3ε can exert neuroprotective effect in ischemic brain through inhibiting neuronal apoptosis.

Autophagy has a beneficial role or adverse effect in cerebral ischemia, depending on various pathological conditions [39], [40]. There is, however, robust evidence that excessive autophagy leads to neuronal death in cerebral ischemia [66], [67], and down-regulation of autophagy can attenuate damage in rat focal ischemic brain and oxygen-glucose deprived astrocytes [52], [46], [47], [68]. In this study, we observed that blockade of autophagy by 3-MA, an autophagy inhibitor, could partly protect brains from I/R-induced Injury, suggesting that inhibition of autophagy seems to be neuroprotective in a rat model of 2 h ischemia and 24 h reperfusion. Recent investigations showed that 14-3-3 family proteins are also regulators of autophagy [41], [42]. However, the role of each 14-3-3 isoform in autophagy is not clear. Herein we report for the first time that pharmacological elevation of 14-3-3ε in the brain attenuates ischemia-induced up-regulation of autophagic activity, which is consistent with the latest reports showing that 14-3-3ε may be involved in autophagy down-regulation in HeLa [41] and gliocytoma U251 cells [69]. Moreover, we found that TAT-14-3-3ε treatment plus rapamycin, a widely used chemical to induce autophagy [70], increased infarct size, and enhanced the autophagic activity when compared with TAT-14-3-3ε-only treatment, suggesting that rapamycin attenuates the neuroprotective effect of TAT-14-3-3ε and inhibition of autophagy is involved in the mechanism of TAT-14-3-3ε. However, TAT-14-3-3ε attenuated LC3B-II upregulation induced by ischemic injury but had no effect on injury-induced upregulation of Beclin-1. Such a different effect of TAT-14-3-3ε on LC3B-II and Beclin-1 may reflect different signal pathways for autophagy in rat ischemia model. 14-3-3ε was shown to bind to both Beclin-1 and intermediate filament proteins [41]. 14-3-3ε is also found to interact with chloride intracellular channel protein 4, which then binds to and retains Beclin-1 [69]. The free Beclin-1 binds to vacuolar sorting protein (VPS34), a class III phosphatidylinositol 3-kinase (PI3K), and forms a core kinase complex which is essential for the induction of autophagy [71]. Since 14-3-3ε strongly binds to Beclin-1, this binding may effectively reduce the levels of free Beclin-1 (available for autophagy induction) and sequester Beclin-1 away from the complex, leading to inhibition of autophagy and thus contributes to the neuroprotective effect of TAT-14-3-3ε. Further investigations are needed to precisely reveal the mechanisms of 14-3-3ε in autophagy.

In summary, TAT-14-3-3ε was prepared and transduced into the brain efficiently, and it exhibited significant protective effect against cerebral I/R injury in rats. The mechanisms underlying the neuroprotection of TAT-14-3-3ε in brain ischemia may be attributed to its inhibition on neuronal apoptosis and autophagy. TAT-14-3-3ε might be a new pharmaceutical target for the treatment of not only stroke but potentially other human injuries and diseases.

## Supporting Information

Table S1 **Primers for synthesis of** ***14-3-3ε*** **gene.**
(DOCX)
