# Supplementary material for: Neuroprotective Effect of TAT-14-3-3ε Fusion Protein against Cerebral Ischemia/Reperfusion Injury in Rats
Source: PLoS One. 2014 Mar 26;9(3):e93334. doi: 10.1371/journal.pone.0093334 (PMC3966873; doi:10.1371/journal.pone.0093334)
Supplement: Table S1 — Primers for synthesis of 14-3-3ε gene. (DOCX) [file pone.0093334.s001.docx]

**Table S1.** **Primers for synthesis of *14-3-3ε* gene.**

| Primers | Oligonucleotides (5’-3’ direction) |
| --- | --- |
| P1 | ATGGATGATCGTGAAGATCTGGTGTATCAGGCGAAACTGGCCGAACAGGC |
| P2 | TTTTTCATTGATTCCACCATTTCATCATAACGTTCCGCCTGTTCGGCCAG |
| P3 | TGGAATCAATGAAAAAAGTGGCGGGCATGGATGTGGAACTGACCGTTG |
| P4 | ACATTTTTATACGCCACAGACAGCAGGTTGCGTTCTTCAACGGTCAGTTC |
| P5 | GTGGCGTATAAAAATGTGATTGGCGCGCGTCGTGCCAG |
| P6 | TTCTTTCTGTTCAATGCTGCTAATAATGCGCCAGCTGGCACGACGCGCGC |
| P7 | TTAGCAGCATTGAACAGAAAGAAGAAAATAAAGGTGGCGAAGATAAACTG |
| P8 | TTCAACCATCTGGCGATATTCACGAATCATTTTCAGTTTATCTTCGCCAC |
| P9 | TATCGCCAGATGGTTGAAACCGAACTGAAACTGATCTGCTGTGATATTC |
| P10 | CTGCCGGAATCAGATGTTTGTCCAGCACATCCAGAATATCACAGCAGATC |
| P11 | CTGATTCCGGCAGCTAATACCGGCGAGTCTAAAGTTTTCTATTATAAAAT |
| P12 | AATTCTGCCAGATAACGATGGTAATCACCTTTCATTTTATAATAGAAAAC |
| P13 | CGTTATCTGGCAGAATTTGCCACCGGTAATGATCGCAAAGAGGCCGCGG |
| P14 | TATCACTCGCCGCTTTATACGCCACCAGGCTATTTTCCGCGGCCTCTTTG |
| P15 | AAGCGGCGAGTGATATCGCAATGACCGAACTGCCGCCGACGCATCCGATC |
| P16 | GTAGAACACTGAAAAATTCAGAGCCAGACCCAGGCGGATCGGATGCGTC |
| P17 | TGAATTTTTCAGTGTTCTACTATGAAATCCTGAATAGCCCGGATCGTGCCTG |
| P18 | CATCATCAAAAGCTGCTTTTGCCAGACGGCAGGCACGATCCGGGCTATTCAG |
| P19 | AGCAGCTTTTGATGATGCGATCGCGGAACTGGATACCCTGAGCGAAGAAAGCTATA |
| P20 | AGATTATCACGCAGCAGCTGCATGATCAGGGTAGAATCTTTATAGCTTTCTTCGCTCAG |
| P21 | GCTGCTGCGTGATAATCTGACCCTGTGGACCAGCGATATGCAGGGTGATGGTGAAGAACA |
| P22 | TTACTGATTTTCATCTTCCACATCCTGCAGCGCTTCTTTATTCTGTTCTTCACCATCACC |
